# Regulatory T cells in transplantation - from preclinical models to clinical study

**DOI:** 10.1186/ar3408

**Published:** 2011-09-16

**Authors:** Kathryn Wood

**Affiliations:** 1 Transplantation Research Immunology Group, Nuffield Department of Surgical Sciences, University of Oxford, John Radcliffe Hospital, Oxford OX3 9DU, UK

## 

After exposure to alloantigen *in vivo *and *in vitro*, alloantigen reactive immunoregulatory activity is enriched in a population of CD4^+ ^T cells that express high levels of CD25, the α chain of the interleukin-2 receptor, and the transcription factor FOXP3. *In vivo*, common mechanisms underpin the activity of CD25^+^CD4^+ ^Treg in adult hosts. We identified a unique role for IFNγ in the functional activity of CD25^+^CD4^+ ^alloantigen reactive Treg during the development of operational tolerance to donor alloantigens *in vivo *that is consistent with observations showing that tolerance to alloantigens cannot be induced in the absence of IFNγ [1]. In order to provide proof of concept data for translation of findings in preclinical models to the bedside, we have demonstrated that human regulatory T cells expanded ex vivo can protect human allografts (skin and vessels) from rejection [2,3].

The identification and characterisation of Treg that can control immune responsiveness to alloantigens has opened up exciting opportunities for new therapies in transplantation.
